# Foraminal Ligaments Tether Upper Cervical Nerve Roots: A Potential Cause of Postoperative C5 Palsy

**DOI:** 10.1055/s-0040-1712982

**Published:** 2020-07-24

**Authors:** Andrew S. Jack, Brooks R. Osburn, Zane A. Tymchak, Wyatt L. Ramey, Rod J. Oskouian, Robert A. Hart, Jens R. Chapman, Line G. Jacques, R. Shane Tubbs

**Affiliations:** 1 Division of Neurosurgery, University of Alberta, Edmonton, Alberta, Canada; 2 Department of Neurological Surgery, University of California San Francisco, San Francisco, California, United States; 3 Complex Spine Surgery, Swedish Neuroscience Institute, Seattle, Washington, United States; 4 Department of Neurosurgery, University of South Florida, Tampa, Florida, United States; 5 Department of Neurosurgery, Tulane University School of Medicine, New Orleans, Louisiana, United States

**Keywords:** C5 palsy, ligament, nerve root, complication, foraminal stenosis, cervical decompression

## Abstract

**Background** Nerve root tethering upon dorsal spinal cord (SC) migration has been proposed as a potential mechanism for postoperative C5 palsy (C5P). To our knowledge, this is the first study to investigate this relationship by anatomically comparing C5–C6 nerve root translation before and after root untethering by cutting the cervical foraminal ligaments (FL).

**Objective** The aim of this study is to determine if C5 root untethering through FL cutting results in increased root translation.

**Methods** Six cadaveric dissections were performed. Nerve roots were exposed via C4–C6 corpectomies and supraclavicular brachial plexus exposure. Pins were inserted into the C5–C6 roots and adjacent foraminal tubercle. Translation was measured as the distance between pins after the SC was dorsally displaced 5 mm before and after FL cutting. Clinical feasibility of FL release was examined by comparing root translation between standard and extended (complete foraminal decompression) foraminotomies. Translation of root levels before and after FL cutting was compared by two-way repeated measures analysis of variance. Statistical significance was set at 0.05.

**Results** Significantly more nerve root translation was observed if the FL was cut versus not-cut, *p* = 0.001; no difference was seen between levels, *p* = 0.33. Performing an extended cervical foraminotomy was technically feasible allowing complete FL release and root untethering, whereas a standard foraminotomy did not.

**Conclusion** FL tether upper cervical nerve roots in their foramina; cutting these ligaments untethers the root and increases translation suggesting they could be harmful in the context of C5P. Further investigation is required examining the value of root untethering in the context of C5P.

## Introduction

Postoperative C5 nerve palsy (C5P) is a common postoperative complication after cervical decompression surgery with a reported incidence ranging between 0 and 30%. 1 2 3 4 Although not a life-threatening injury, its occurrence can be quite disabling for patients with neurotization salvage procedures being the only treatment option for those not demonstrating functional recovery. 5 6 Many factors have been associated with its occurrence including foraminal stenosis, surgical approach (anterior vs. posterior decompression), ossification of the posterior longitudinal ligament, among others. 3 4 With the exception of direct nerve root injury identified at the time of surgery, none of these factors have been proven to be causative. As such, there have been several attempts to explain the pathophysiological mechanism underlying the occurrence of C5P, including direct nerve root injury (surgical trauma), nerve root ischemia, reperfusion injury postdecompression, postoperative plexitis/neuritis, preoperative and subsequent postoperative SC rotation, nerve root tethering, and traction injury upon dorsal SC migration. 2 3 7 8 9 10 The last-named form the basis of this study.

Elaborating on the nerve root traction hypothesis, we propose that another factor contributing to postoperative C5P is tethering of the nerve root within the vertebral foramen at the C5 level. The C5 nerve root is anatomically unique in that it has shorter rootlets than other nerve roots, 11 has the most horizontal course to the vertebral foramen, 12 is the smallest nerve root, 13 and has more numerous and more robust foraminal ligaments (FL) associated with its intervertebral foramen. 13 14 15 Its unique anatomical course in combination with its having more numerous and thicker ligamentous support at the foraminal level predisposes the C5 nerve root to traction injury upon dorsal SC migration postdecompression.

Posterior cervical decompression results in dorsal migration of the SC upon removal of the overlying compressive pathology. 2 7 10 Once the SC migrates posteriorly, it is plausible that the FL (comprised of both intraforaminal ligaments [IFL]connecting the perineurium to the periosteum and extraforaminal ligaments connecting the nerve sheath to the uncovertebral and facet joints) 15 16 17 18 19 20 21 also contribute to nerve root tethering resulting in a traction injury. Our aim here is to test this hypothesis by anatomically comparing the amount of C5 and C6 intraforaminal nerve root translation postcervical decompression that occurs both before and after release of the FL. In an attempt to corroborate this FL tethering effect on the nerve roots, examine the potential protective effects of the FL, and simulate intraoperative positioning, we also measure the amount of translation of these nerve roots with downward shoulder displacement before and after ligamentous untethering.

## Materials and Methods

**Fig. 1:**
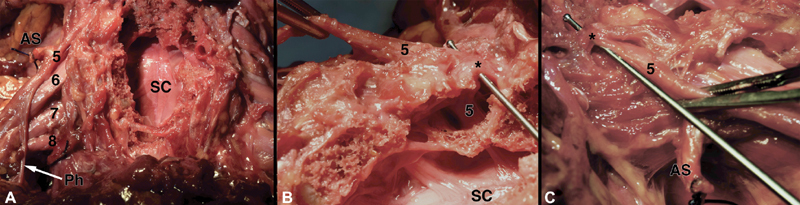
( **A** ) Exposure of cervical spinal cord after corpectomy, intradural, and extradural nerve roots (cervical roots labeled 5–8), and bilateral supraclavicular brachial plexus. ( **B** ) Medial-to-lateral visualization of the right C5 intradural and extradural nerve root from ( **A** ) (held with forceps in top left of the panel) with a ball-tip probe passing over the nerve root and under a FL (marked by *). ( **C** ) Lateral-to-medial visualization of ( **B** ) (FL marked by *). AS, anterior scalene (tied off); FL, foraminal ligament; Ph, phrenic nerve; SC, spinal cord.

Six cadaveric dissections were performed (mean age at death = 70 years). Institutional review board/ethics committee approval was not necessary as this was a cadaveric study. Intradural nerve roots were exposed with a surgical microscope via corpectomy of C4–C6 followed by durotomy to visualize the SC, nerve rootlets, and intradural roots. Extradural nerve roots were exposed via a standard supraclavicular brachial plexus approach ( Fig. 1A ). Metallic pins were inserted into the foraminal C5 and C6 roots and each of their respective adjacent anterior bony foraminal tubercle. The SC has been shown to translate up to and in excess of 5 mm after posterior decompression. 7 10 To simulate this, the SC and intradural nerve roots at C5 and C6 were manually displaced 5 mm dorsally. Upon dorsal displacement of the SC and its rootlets, any corresponding intraforaminal nerve root translation was then measured using calipers as the distance between the metallic pins both before and after cutting the FL ( Fig. 1B, C ) using the surgical microscope as previously described. 1 To corroborate the potential role that FL may have in nerve root tethering and simulate their role in nerve root tethering during intraoperative positioning (as well as their potential protective role), root translation was also measured using calipers during downward shoulder displacement as previously described during intraoperative prone positioning. 1 For each translational nerve root measurement, two of the authors measured and recorded the amount of nerve root translation independently and in blinded-fashion with an average of the two measurements being used for analysis. Finally, to determine the clinical feasibility of untethering the nerve roots, we performed a standard and an extended foraminotomy on a cadaveric specimen after placement of C1–T2 instrumentation (Mountaineer cervicothoracic instrumentation, Dupuy-Synthes, Raynham, Massachusetts, United States) and laminectomy decompression. We then began to lyse the FL, continuing out laterally until the nerve root was completely free. To determine the difference in nerve root untethering between a standard and an extended foraminotomy, we also performed a posterior paramedian dissection to the brachial plexus 22 and identified the C5 and C6 nerve roots. Lateral traction was placed on the C5 nerve root, and we looked for any corresponding nerve translation in the nerve root foramen and spinal canal more proximally. Two-way repeated measures analysis of variance with Bonferroni's post hoc statistical testing using SPSS (v23.0, IBM Corporation, Armonk, New York, United States) computer software was used to compare translation of the different root levels before and after FL cutting. Statistical significance was set at 0.05.

## Results

### Nerve Root Translation before and after Ligamentous Cutting

**Fig. 2:**
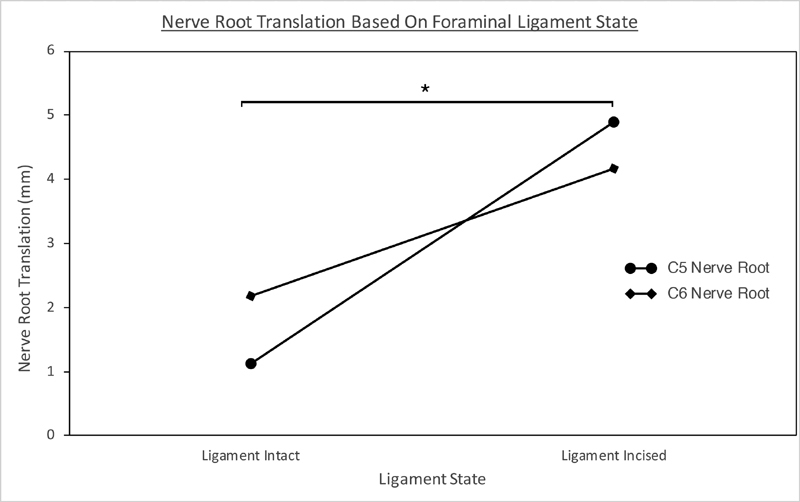
The effect of foraminal ligament cutting on nerve root translation. There was a significant difference between nerve root translation pre- and postligament incision (denoted by *) for both C5 and C6 nerve roots.

The FL cutting increased translation at both C5 and C6 nerve roots ( Table 1 ). Mean C5 nerve root translation was 1.12 mm (±0.75 mm) before (ranging from 0.25 to 2.19 mm) and 4.89 mm (±0.97 mm) after (ranging from 4.75 to 5.00 mm) ligament cutting. Mean C6 translation was 2.17 mm (±1.18 mm) before (ranging from 0.55 to 3.52 mm) and 4.16 mm (±0.48 mm) after (ranging from 3.68 to 4.75mm) ligament cutting. There was a significant interaction in translation between ligament status (cut vs. not-cut) and root level, F(1, 5) = 23.89, *p* < 0.01. Significantly more nerve root translation was seen if the ligaments were cut versus not-cut ( Fig. 2 ), F(1, 5) = 47.35, *p* < 0.01. However, there was no difference between root levels, F(1, 5) = 1.18, *p* = 0.33.

**Table 1:** Mean nerve root translation before and after ligamentous release, with and without downward shoulder displacement

Nerve root	Mean translation (ligament intact) ± standard deviation (mm)	Mean translation (ligament incised) ± standard deviation (mm)	*P* -value
C5	1.12 ± 0.75	4.89 ± 0.97	<0.01
C6	2.17 ± 1.18	4.16 ± 0.48	<0.01
C5 with downward shoulder displacement	0.54 ± 0.51	6.90 ± 2.64	<0.01
C6 with downward shoulder displacement	0.37 ± 0.34	5.05 ± 1.30	<0.01

### Nerve Root Translation upon Downward Shoulder Displacement before and after Ligamentous Cutting

The FL cutting increased translation upon downward shoulder displacement at both C5 and C6 nerve roots ( Table 1 ). Mean C5 nerve root translation was 0.54 mm (±0.51 mm) before (ranging from 0.05 to 1.43 mm) and 6.90 mm (±2.64 mm) after (ranging from 3.54 to 9.80 mm) ligament cutting. Mean C6 nerve root translation was 0.37 mm (±0.34) before (ranging from 0.00 to 1.19 mm) and 5.05 mm (±1.30 mm) after (ranging from 3.36 to 6.40 mm) ligament cutting. There was a significant interaction in translation between FL status (cut vs. not-cut) and root level upon downward shoulder displacement, F(1, 5) = 6.91, *p* < 0.05. Significantly more root translation with downward shoulder displacement was seen if the ligaments were cut versus not-cut, F(1, 5) = 29.87, *p* < 0.01. There was also a significant difference between root levels, F(1, 5) = 10.47, *p* = 0.02.

### Clinical Feasibility of an Extended Foraminotomy

**Fig. 3:**
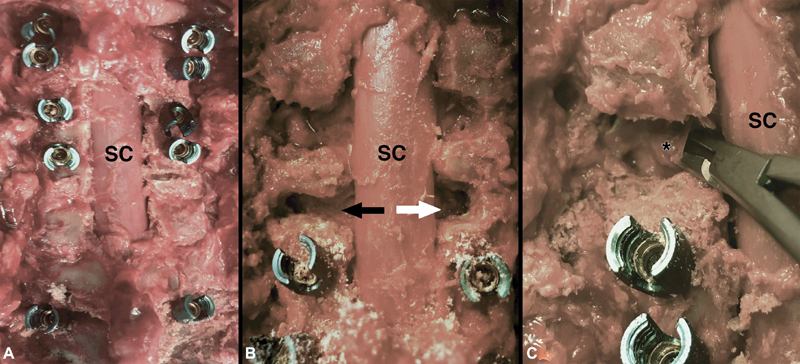
( **A** ) Exposure of the cervical spinal cord after C1–T2 instrumentation and laminectomy demonstrating the clinical feasibility of nerve root untethering. ( **B** ) Enlarged view of both a standard foraminotomy (white arrow, right-side) and “extended foraminotomy” (black arrow, left-side). ( **C** ) Enlarged view of the “extended foraminotomy” from ( **B** ) at the beginning of the nerve root untethering via foraminal ligament (marked by *) cutting. SC, spinal cord.

As an adjunct to this study, we investigated the clinical feasibility of an extended foraminotomy, including complete lateral “unroofing” of the foramen through drilling of the lateral mass ( Fig. 3 ). For this, we performed a posterior cadaveric dissection, including laminectomy decompression and C1–T2 instrumentation implantation ( Fig. 3A ). We then performed a standard foraminotomy on one side and an extended foraminotomy on the other (right and left sides of Fig. 3B , respectively). After performing the extended foraminotomy, lysis of the FL around the nerve root was performed and continued laterally until neurolysis was completed out to the lateral aspect of the foramen. To corroborate the nerve root translation results above, a posterior paramedian brachial plexus exposure as described by Dubuisson et al was completed and lateral traction placed on the extraforaminal C5 nerve root. 22 With lateral traction, little translation of the nerve was visualized on the standard foraminotomy side, whereas there was an almost identical amount of corresponding translation on the side of the extended foraminotomy that was transmitted back to the foraminal portion of the nerve root and its rootlets. This supports the notion of the FL acting as a foraminal nerve root tether and being potentially harmful in the context of postoperative C5P. This also lends credence to the idea that standard foraminotomy is not sufficient to prevent FL nerve root tethering that could contribute to postoperative C5P, and the feasibility of an extended foraminotomy for neurolysis.

## Discussion

Our results demonstrate that cutting the FL results in detethering of C5 and C6 nerve roots. This allows the nerve roots to move freely in conjunction with the SC upon, for example, its dorsal migration after posterior cervical decompression or SC de-rotation postdecompression. This phenomenon of posterior SC shift and cord de-rotation postdecompression, in addition to the width of the cervical foramina have been shown to be a risk factors for the development of postoperative C5P. 2 5 23 24 25 26 27 28 29 30 31 These results are also supported by studies showing that foraminotomy at the C4–C5 level is associated with a lower incidence of C5P. 32 33 Although the development of a C5P is likely to be a final common clinical pathway for any sort of insult to the C5 nerve perioperatively (explaining the diversity in both clinical symptomatology and postoperative onset), the nerve root tethering/traction hypothesis helps explain C5P occurrence in the setting of foraminal stenosis and/or dorsal SC migration and de-rotation postdecompression. In fact, in a recent systematic review by Jack et al employing stringent inclusion criteria to elucidate risk factors for C5P due to a common tethering pathophysiology, foraminal stenosis and preoperative SC rotation were both found to be C5P risk factors. 31 Furthermore, the delay in onset of C5P symptoms after decompression (postoperative days 2–7) 1 26 also seems to support this hypothesis: posterior SC shift and de-rotation, as well as C5P development can take time to occur. Moreover, some patients without foraminal stenosis and patients undergoing anterior surgical decompression may experience a C5P complication. 26 27 28 After an anterior approach, FL may tether and strain the nerve roots upon indirect foraminal decompression with insertion of strut graft.

Several explanations have been offered as to why C5 seems particularly predisposed to injury after cervical decompression. The rootlets at C5 are shorter than at other cervical levels, C5 is one of the only nerve roots with a proximal intraforaminal branching pattern due to the dorsal scapular nerve take-off (potentially contributing to intraforaminal tethering), and C5 is the smallest nerve root. 11 13 34 Because of these particular anatomical features, C5 nerve rootlets would likely bear the brunt of the strain upon dorsal SC migration and de-rotation; furthermore, they would shield other nerve roots from being stretched. The C5 and C6 nerve roots also have the most supporting ligaments, as well as the thickest. 13 14 15 Arslan et al classified the cervical intraforaminal and extraforaminal ligaments, characterizing the latter as transforaminal or radiating. Transforaminal ligaments (TFL) traverse the intervertebral foramen and loosely attach the spinal nerve root to adjacent structures, but the radiating ligaments are thicker, stronger, and firmly attached to the nerve root. They can be further subdivided into ventral superior and inferior, and dorsal superior and inferior. 13 In their anatomical study, Akdemir et al 16 found the IFL to emanate from the nerve sheath perineurium and attach to the periosteum and TFL. Together, these FLs center the nerve root in the foramen, reducing friction, and improving blood flow and electrophysiological transmission. 15 16 17 18 19 20 21 35 36 37 38 39 The role of these cervical FLs in holding and maintaining the central position of the nerve roots in their respective foramen and the result of this tethering is highlighted by physiological studies showing a 70% decrease in action potential amplitude with only 6% nerve root strain and complete conduction block at 12% strain. 40 Moreover, blood flow is decreased at 8% strain and intraneural circulation ceases completely at 15%. As such, the increased nerve root strain due to tethering from the FL after posterior SC shift likely impairs conduction through the nerve and results in C5P signs and symptoms. Finally, the C5 and C6 nerve roots have the most numerous and robust FL. The strength of the FL at these levels and their ability to tether their respective nerve roots is also emphasized upon considering that these levels have the lowest incidence of avulsed nerve roots (in comparison to lower cervical and upper thoracic nerve roots which have the highest incidence of nerve root avulsion and least numerous FL). 41 42 43

Others have argued that cervical FL also become pathological upon calcification, fibrotic degeneration, and hypertrophy, resulting in nerve root entrapment and pain. 44 45 46 Similarly, our results support the view that these ligaments could be pathological in the context of postoperative C5P (resulting from intraforaminal nerve root tethering). Moreover, as foraminal stenosis has been found to be an independent risk factor for postoperative C5P upon systematic review and foraminotomy has been found to lower its incidence in some studies, 24 26 31 32 33 47 48 49 it is plausible that these FL act in conjunction with other degenerative causes of foraminal stenosis to tether the nerve root and lead to C5P. Although our paper demonstrates that cervical FLs tether the C5 and C6 nerve roots in their foramina potentially contributing to C5P and that extended foraminotomy allows for neurolysis and ligamentous release, further research is required. The role of these ligaments in the development of C5P is still speculative. As previously suggested, the development of a postoperative C5P is likely to be due to a combination of factors, the state of the cervical FL being merely contributory or correlative, not necessarily causative. Further studies, including investigation and anatomical description of the intraforaminal dorsal scapular nerve branch point as a potential cause of tethering, as well as development of an animal model for C5P is currently underway. *In vivo* animal feasibility studies examining the electrophysiological and clinical result (both intraoperative and postoperative) of nerve root traction before and after foraminal root neurolysis will be helpful in elucidating the pathophysiology of traction C5P. These studies will also serve as a first-step in translation of a potential treatment option if feasible.

This cadaveric study has obvious, inherent limitations, and extrapolation to an *in vivo* patient scenario requires further translational study. As mentioned, given the dual function of the FL (harmful in the context of postoperative C5P, yet also potentially protective in preventing distal forces being transmitted proximally upon, for example, traction-avulsion injuries), 15 20 36 our anatomical results will require further investigation in an *in vivo* animal pilot study prior to clinical implementation. However, the results and the potential role of the FL in C5P highlighted in this study help to lay a foundation on which these studies may occur. We used fixed specimens, which can cause anatomical shrinking and stiffening, biasing our measurements. Furthermore, this study was limited by both the age of the cadavers and the number of cadaveric specimens used. However, despite the small numbers, there was a significant difference, emphasizing the possible effect of nerve root untethering in the context of C5P. Furthermore, it is often older patients who undergo cervical decompressive surgery, suggesting the particular relevance of these results to this population. The feasibility/applicability of nerve root untethering from an anterior approach was also not attempted and would likely be much more difficult with a higher complication profile given the location of the vertebral artery. Assessment of this issue and feasibility of this approach in animal studies is currently underway. Moreover, application of the extended foraminotomy to untether the C5 nerve root in a patient would also necessitate instrumented stabilization, potentially limiting its use to those patients at high risk for C5P and requiring cervical fusion at the index surgery or those patients developing a severe postoperative C5P not initially improving with conservative management (both previously shown to be prognostic factors for C5P recovery). 50 Further study is required to examine the role and timing of extended foraminotomy, its *in vivo* applicability, as well as its comparison with current therapeutic strategies including delayed neurotization procedures.

## Conclusion

To our knowledge, this is one of the first studies to consider the role of the cervical FL in the development of postoperative C5P. Our results suggest that FL play an integral role in foraminal nerve root tethering and preventing the translation of the C5 and C6 nerve roots, potentially contributing to C5P. Understanding and considering the nerve root tethering that these ligaments cause after cervical decompression could help clinicians in both avoiding postoperative C5P and treating patients who have developed it. This study helps establish a foundation for further investigation pertaining to the role that FL may play in C5P.
